# Incidence of immunization errors in the state of Minas Gerais, Brazil: a cross-sectional study, 2015-2019

**DOI:** 10.1590/S2237-96222022000300008

**Published:** 2022-11-04

**Authors:** Deborah Amaral Donnini, Carlos Miguel Bolognani Silva, Josianne Dias Gusmão, Fernanda Penido Matozinhos, Roberta Barros Silva, Gabriela Gonçalves Amaral, Eliete Albano de Azevedo Guimarães, Valéria Conceição de Oliveira

**Affiliations:** 1 Universidade Federal de São João del-Rei, Programa de Pós-Graduação em Enfermagem, Divinópolis, MG, Brazil; 2 Secretaria de Estado da Saúde de Minas Gerais, Superintendência de Vigilância Epidemiológica, Belo Horizonte, MG, Brazil; 3 Universidade Federal de Minas Gerais, Programa de Pós-Graduação em Enfermagem, Belo Horizonte, MG, Brazil; 4 Universidade de São Paulo, Programa de Pós-Graduação em Enfermagem em Saúde Pública, Ribierão Preto, SP, Brazil

**Keywords:** Vaccination, Drug-Related Side Effects and Adverse Reactions, Medication Errors, Patient Safety, Primary Health Care, Descriptive Epidemiology

## Abstract

**Objective:**

To evaluate the incidence of immunization errors in the public health service of the state of Minas Gerais, Brazil.

**Methods:**

This was a cross-sectional study, based on errors reported on the National Immunization Program Information System between 2015 and 2019. A descriptive analysis and calculation of the incidence for the state's health macro-regions were performed.

**Results:**

A total of 3,829 notifications were analyzed. Children younger than 1 year old were the most affected (39.1%) and the intramuscular route accounted for 29.4% of the errors. The most frequently reported error was administration of vaccines outside minimum and maximum recommended ages (37.7%). There was a higher incidence of errors in Vale do Aço (26.5/100,000) and Triângulo do Norte (22.6/100,000) macro-regions.

**Conclusion:**

Immunization errors showed a heterogeneous incidence among the macro-regions of the state of Minas Gerais, between 2015-2019, and the administration of vaccines outside minimum and maximum recommended ages was the most frequently reported error.

**Study contributions**

**Main results**

There was an increase in the incidence of immunization errors in all health macro-regions of Minas Gerais state. The most frequently reported type of error was vaccine doses administered outside the recommended age; results point to underreporting of these errors.

**Implications for services**

The increased incidence of immunization error may compromise vaccination coverage, in addition to an increased risk of adverse events following vaccination. Investing in permanent education of workers and risk management are strategies to reduce these errors.

**Perspectives**

To advance in knowledge of professionals about the practice in the vaccination room and analyze the factors that influence the notification and underreporting of immunization errors in different health macro-regions of Minas Gerais state.

## Introduction

Vaccination is a health strategy with great effectiveness. Due to its action in disease prevention, it avoids millions of deaths per year and increases life expectancy.^1^ As with all medicines administration, vaccination errors are known to occur.^2^ Immunization errors are preventable as they are consequences of attitudes or procedures that have not been followed accordingly.^3^

According to the rules of the National Immunization Program (*Programa Nacional de Imunizações* - PNI), immunization errors may cause reduction or lack of the expected effect of vaccines, in addition to adverse events following immunization (AEFI).^3^ These errors may also have a negative impact on the population, interfering in the follow-up of the vaccination schedule, reducing vaccination coverage rates and jeopardizing the control of vaccine-preventable diseases,^4^ ^-^ ^6^ in addition to generating direct and indirect costs to health services.^7^ ^-^ ^8^

In the last ten years, the international^5^ ^,^ ^9^ ^-^ ^10^ and national literature^4^ ^,^ ^6^ have pointed to an increase in notifications of immunization errors. A study conducted between 2001 and 2016, aiming to describe the characteristics of vaccination errors using a European database, identified an increase in the number of notifications of vaccine errors, from 0.4%, in 2001, to 4% in 2016.^10^ In the United States, between 2000 and 2013, the Vaccine Adverse Event Reporting System also observed an increase in immunization error notifications from 1%, in 2000, to 15% in 2013.^5^

With regard to Brazil, a study conducted in the state of Paraná on records of AEFI due to immunization errors, focused on the period from 2003 to 2013, identified an increase of 0.184 in the incidence rate per 100,000 doses administered. The mean value estimated by the same study for the period 2014-2018 ranged from 2.5 (2014) to 3.3 (2018) AEFI due to immunization errors per 100,000 doses administered.^4^ In the state of Goiás, the overall incidence rate of errors was 4.1/100,000 doses administered, and the highest incidence rates were related to the human rabies vaccine, the human papillomavirus vaccine and the triple viral vaccine; the incidence rate of errors regarding AEFI was 0.45/100,000 doses administered.^6^ In the state of Minas Gerais, a study conducted between 2015 and 2019, aimed at analyzing immunization errors in pregnant women, according to the absence and presence of AEFI, found an incidence of 2.07/100,000 doses administered, showing errors and some adverse events.^11^

In Brazil, records of immunization errors in individuals vaccinated within the public health network are made available in the Adverse Event Following Immunization Surviellance Information System (*Sistema de Informação de Vigilância de Eventos Adversos Pós-Vacinação* - SI-EAPV).^3^ In order to support the completion of information, the SI-EAPV has its own notification/investigation form, in which the information is inserted to characterize the error and provide instructions on the conduct to be adopted in case of AEFI and in the face of the vaccination schedule.

Given the increase in scientific literature on immunization errors^4^ ^,^ ^6^ ^,^ ^10^ and the importance of knowledge about thier occurrence for assertive decision-making in health services and practices, we believe that this study can provide a comprehensive understanding of the occurrence of immunization errors in the coming years, in the state of Minas Gerais, the second most populous state with the largest number of municipalities in Brazil.^12^ ^,^ ^13^

The aim of this study was to evaluate the incidence of immunization errors in the public health service in the state of Minas Gerais, Brazil, between 2015 and 2019.

## Methods

This was a descriptive cross-sectional study based on the notifications of immunization errors recorded in the AEFI database of the PNI Information System (SI-PNI), in Minas Gerais, from January 1, 2015 to December 31, 2019. We accessed this database, made available by the State Department of Health of Minas Gerais, from March to November 2020.

Minas Gerais had an estimated population of 21,411,923 inhabitants in 2021, and a human development index (HDI) of 0.731 in 2010.^12^ Based on demographic, socioeconomic, geographical, sanitary and epidemiological characteristics, the state territory is divided into 14 macro-regions for health care planning; these macro-regions are subdivided into 89 microregions, covering a total of 853 municipalities. Central is the most densely populated macro-region, where the capital of Minas Gerais is located, which is the most populated city, with 31.7% of the total population of the state; and Jequitinhonha is the least populated macro-region.^13^

The study population was comprised of all individuals who recieved any type of immunobiological agent within the public health system, experienced any type of immunization error and had this error registered on the SI-EAPV.

The outcome variable of the study was the occurrence of immunization errors classified according to the form for notification/investigation of AEFI associated with the use of vaccines, serum or immunoglobulin (handling/conservation errors; dilution errors; administration of vaccines outside minimum and maximum recommended ages; inadequate interval between doses/vaccine; administration errors; type of immunobiological product used; expired immunobiological product; other).^3^ Defined doses for routine vaccines, recommended ages, minimum intervals between doses and minimum and maximum ages for vaccine administration, according to the Brazilian PNI, are shown in Box 1.

**Box 1:** Definition of doses by vaccines, recommended ages, minimum intervals between doses and minimum and maximum ages for administration, Brazil, 2022

Vaccine	Recommended age	Recommended minimum interval between doses	Maximum age
BCG^a^	At birth	Single dose	4 years 11 months 29 days
HB^b^ first dose	At birth	30 days	1 month
OHVR^c^	2 and 4 months	30 days	1^st^ dose: until 3 months 15 days
			2^nd^ dose: until 7 months 29 days
Pentavalent^d^ (DTP + HB^b^ + Hib)	2, 4 and 6 months	30 days between the 1^st^, 2^nd^ and 3^rd^ dose of pentavalent. The 3^rd^ dose should not be administered before 6 months old	6 years 11 months 29 days
Polio (IPV^e^)	2, 4 and 6 months	30 days between the 1^st^, 2^nd^ and 3^rd^ dose of IPVe. 6 months between the 3^rd^ dose of IPV^e^ and the 1^st^ booster dose of OPV^f^	4 years 11 months 29 days
Polio (OPV^f^)	15 months and 4 years	6 months between the 3^rd^ dose of IPV^e^ and the 1^st^ booster dose of OPV^f^. 6 months between the 1^st^ and 2^nd^ booster dose of OPV^f^	4 years 11 months 29 days
Pn10^g^	2, 4 and 12 months	30 days between 1^st^ and 2^nd^ dose. 60 days between the 2^nd^ dose and the booster dose at 12 months	4 years 11 months 29 days
MenC^h^	3, 5 and 12 months	30 days between 1^st^ and 2^nd^ dose. 60 days between the 2^nd^ dose and the booster dose at 12 months	4 years 11 months 29 days
YF^i^	9 months and 4 years*	30 days between doses of YF^i^	-
MMR^j^	12 months and 15 months***	30 days of interval of YF^i^* vaccine	-
HA^k^	15 months	-	4 years 11 months 29 days
DTP^l^	15 months and 4 years	6 months between the 3^rd^ dose of pentavalent and the 1^st^ booster dose of DTPl. 6 months between the 1^st^ and 2^nd^ booster dose of DTP^l^	6 years 11 months 29 days
VZV^m^	15 months and 4 years	30 days of interval of MMR^j^ and YF*** vaccines	6 months 11 months 29 days
HPV^n^	Boys: from 11 to 14 years and girls: from 9 to 14 years	2 doses with minimum interval of 6 months	Under 15 years old
ACWY^o^	11 and 12 years	Single dose	-
DT^p^	From 7 years old	3 doses with recommended interval of 60 days and minimum of 30 days	-
dTpa^q^	Pregnant women	1 dose at each pregnancy (from the 20 weeks pregnant)	-

a) BCG: Bacillus Calmette-Guérin vaccine; b) HB: hepatitis B vaccine; c) OHVR: oral human rotavirus vaccine; d) Pentavalent (DTP+HB+Hib): diphtheria, tetanus, pertussis, hepatitis B adsorbed vaccine (recombinant) and *Haemophilus Influenzae* B (conjugate); e) Poliomyelitis (IPV): injectable, trivalent inactivated poliovirus vaccine; f) Poliomyelitis (OPV): bivalent attenuated oral poliovirus vaccine (OPV); g) Pn10: pneumococcal conjugate vaccine 10-valent; h) MenC: meningococcal C conjugate vaccine; i) YF: yellow fever vaccine; j) MMR: measles, mumps and rubella virus vaccine; k) HA: hepatitis A vaccine; l) DTP: triple bacterial vaccine (diphtheria, tetanus and pertussis combination vaccine); m) VZY: attenuated varicella vaccine; n) HPV: human papillomavirus vaccine 6, 11, 16 and 18 (recombinant); o) ACWY: ACWY meningococcal vaccine (conjugate); p) DT: adult diphtheria and tetanus adsorbed vaccine; q) dTpa: adult diphtheria, tetanus and pertussis adsorbed vaccine (acellular). Notes: * People from 5 to 59 years of age: one single dose should be administered; ** People from 5 to 29 years of age who are not vaccinated or with an incomplete vaccination schedule should recieve or complete the two-dose schedule of triple viral, with a minimum interval of 30 days between doses. People from 30 to 59 years of age who are not vaccinated should recieve a triple viral dose; ***When they are not administered simultaneously and with a 30-day interval between yellow fever and triple viral vaccines for children under 2 years of age.

The exposure variables were those existing in the immunization error notification form: age group (years: up to 1; 1 to 4; 5 to 9; 10 to 19; 20 to 59; 60 or over); route of administration (intramuscular; subcutaneous; oral; intradermal; not specified); type of event (immunization error without AEFI; immunization error with AEFI); year of immunization error notification (2015; 2016; 2017; 2018; 2019); health macro-region (Sul; Centro Sul; Centro; Jequitinhonha; Oeste; Leste; Sudeste; Norte; Noroeste; Leste do Sul; Nordeste; Triângulo do Sul; Triângulo do Norte; Vale do Aço).

Before analyzing the data, duplicate records were excluded. A descriptive analysis of the data was performed, including the frequency distribution and differences between proportions, according to demographic characteristics (age group), type of error and route of administration. In order to calculate the incidence rate of immunization errors, per 100,000 doses administered, the total number of errors reported on SI-EAPV (numerator) and the number of doses administered in the period (denominator), by health macro-region, were considered. In the state of Minas Gerais, from 2015 to 2019, 57,289,277 records of vaccine doses administered and 3,866 notifications of immunization errors were found.^14^

A database was built using Microsoft Excel 2010. The Statistical Software Package (Stata), version 14.0, was used for data analysis. 

This research is part of a larger project entitled "Evaluation of immunization errors and intervention proposal", approved by the Human Research Ethics Committee of the Campus Centro-Oeste Dona Lindu/Universidade Federal de São João del-Rei (CEPCO/UFSJ), on January 31, 2020: Opinion No. 3.817.007; Certificate of Submission for Ethical Appraisal (CAAE) No. 23888819.9.0000.5545.

## Results

In the state of Minas Gerais, between 2015 and 2019, 3,866 notifications of immunization errors were identified on the SI-EAPV database. 37 duplicate records were excluded, and a total of 3,829 notifications remained. Of the 853 municipalities in Minas Gerais, 332 (38.9%) reported at least one type of error.

Regarding the characteristics of the 3,829 notifications analyzed, it could be seen that females accounted for (58.1%) of the reported cases. Among the most affected age groups, children under 4 years of age (58.3%) stood out, showing a higher proportion for those under 1 year old (39.1%), followed by those between 20 and 59 years old (20.0%). Intramuscular and subcutaneous routes accounted for 29.4% and 27.8% of the reported errors, respectively. It could be seen that the most frequently reported immunization error was the administration of vaccine outside minimum and maximum recommended ages (37.5%). The prevalence of vaccines that are not recommended during pregnancy was 10.8%. Among the notifications analyzed, it is worth highlighting that in 1,175 (30.7%), the route of administration related to immunization error was not specified (Table 1).

**Table 1:** Characteristics of notifications of immunization errors (n = 3,829), Minas Gerais state, Brazil, 2015-2019

Variable	n	%
**Age group (in years)**		
< 1	1,497	39.1
1-4	735	19.2
5-9	213	5.6
10-19	435	11.4
20-59	767	20.0
≥ 60	183	4.7
**Route of administration**		
Intramuscular	1,127	29.4
Subcutaneous	1,065	27.8
Oral	304	7.9
Intradermal	158	4.2
Not specified	1,175	30.7
**Immunization errors**		
Handling errors	4	0.1
Dilution errors	79	2.1
Vaccine administered outside the recommended age	1,435	37.5
Inadequate interval between doses/vaccines	270	7.1
Administration errors	131	3.4
Type of immunobiological product used	313	8.2
Expired immunobiological product	246	6.4
Repeated doses^a^	231	6.0
Vaccine not recommended during pregnancy^a^	414	10.8
Other	706	18.4

a) They are not included in the classification, according to the form for notification/investigation of adverse events following vaccination associated with the use of vaccine, serum or immunoglobulin.

Regarding the incidence rate by type of immunization error, it could be seen that vaccines administered outside minimum and maximum recommended ages accounted for the most incident error (2.6/100,000 doses administered), followed by administration of vaccines that are not recommended during pregnancy (0.7/100,000 doses administered). The type of immunobiological product used and the inadequate interval between doses/vaccine showed an incidence of 0.6 and 0.5 per 100,000 doses administered, respectively. Expired and repeated vaccine doses showed an incidence rate of 0.4/100,000 doses administered each. When we added administration, dilution and handling errors, the incidence rate found was 0.4 per 100,000 doses administered (data are not shown in the tables).

Table 2 shows the incidence of immunization errors by health macro-region of Minas Gerais. The highest incidence of errors was found in Vale do Aço macro-region, at an incidence rate of 26.5 errors for every 100,000 doses administered, followed by the Triângulo do Norte, with an incidence rate of 22.6 errors for every 100,000 doses administered. On the other hand, the health macro-regions with the lowest incidence of errors reported were: the Noroeste, with 1.6 error per 100,000 doses administered, and the Nordeste, with 1.8 error per 100,000 doses administered. It could be seen that 2019 was the year with the highest incidence of notifications in most health macro-regions of the state, except for the Oeste and Jequitinhonha macro-regions, which showed a higher number of notifications in 2018, and Triângulo do Norte in 2017.

**Table 2:** Incidence of immunization errors (n = 3,829) by health macro-region and year of notification, Minas Gerais state, Brazil, 2015-2019

Health macro-region of Minas Gerais state		2015	2016	2017	2018	2019	Total
Sul	DA^a^	1,358,770	1,172,935	1,827,014	1,676,080	1,364,739	7,399,538
	ie^b^	36	61	97	138	133	465
	IR^c^	2.6	5.2	5.3	8.2	9.7	6.2
Centro Sul	DA^a^	373,400	320,636	568,321	609,088	367,798	2,239,243
	ie^b^	3	3	15	29	116	166
	IR^c^	0.8	0.9	2.6	4.7	31.5	7.4
Centro	DA^a^	3,313,133	2,823,093	4,460,980	3,867,078	3,181,872	17,646,156
	ie^b^	84	115	204	173	230	806
	IR^c^	2.5	4.0	4.5	4.4	7.2	4.5
Jequitinhonha	DA^a^	227,336	160,669	284,157	197,085	172,382	1,041,629
	ie^b^	5	12	18	15	7	57
	IR^c^	2.1	7.4	6.3	7.6	4.0	5.5
Oeste	DA^a^	570,380	525,381	822,539	717,814	619,164	3,255,278
	ie^b^	16	33	55	97	68	269
	IR^c^	2.8	6.2	6.6	13.5	10.9	8.3
Leste	DA^a^	365,302	303,120	576,272	341,185	346,025	1,931,904
	ie^b^	3	2	10	10	16	41
	IR^c^	0.8	0.6	1.7	2.9	4.6	2.1
Sudeste	DA^a^	796,230	702,297	1,273,795	990,293	621,976	4,384,591
	ie^b^	14	21	26	43	80	184
	IR^c^	1.7	2.9	2.0	4.3	12.8	4.2
Norte	DA^a^	934,762	714,015	1,173,550	828,613	732,807	4,383,747
	ie^b^	44	26	35	33	88	226
	IR^c^	4.7	3.6	2.9	3.9	12.0	5.2
Noroeste	DA^a^	362,110	275,505	420,099	393,825	323,306	1,774,845
	ie^b^	4	3	7	5	9	28
	IR^c^	1.1	1.0	1.6	1.2	2.7	1.6
Leste do Sul	DA^a^	302,559	260,432	614,566	415,056	365,493	1,958,106
	ie^b^	13	11	15	34	30	103
	IR^c^	4.2	4.2	2.4	8.1	8.2	5.3
Nordeste	DA^a^	394,653	352,008	770,705	434,198	390,461	2,342,025
	ie^b^	4	5	9	7	18	43
	IR^c^	1.0	1.4	1.1	1.6	4.6	1.8
Triângulo do Sul	DA^a^	369,329	347,976	531,873	438,174	393,480	2,080,832
	ie^b^	37	20	73	72	73	275
	IR^c^	10.0	5.7	13.7	16.4	18.5	13.2
Triângulo do Norte	DA^a^	734,883	633,674	832,001	1,001,145	725,908	3,927,611
	ie^b^	13	91	185	167	432	888
	IR^c^	1.8	22.2	59.5	1.8	22.2	22.6
Vale do Aço	DA^a^	259,760	231,004	209,691	200,028	145,226	1,045,709
	ie^b^	20	13	97	30	117	277
	IR^c^	7.6	5.6	46.2	14.9	80.5	26.5

DA: Number of records of doses administered; b) ie: Number of records of immunization errors; c) IR: Incidence rate of immunization errors per 100,000 doses administered.

The incidence rate of immunization errors with AEFI (323 cases) was 0.56/100,000 doses administered (data are not shown in the tables). The most frequently reported AEFIs due to immunization errors were localized reactions (80.8%), and, in some notifications, more than one local reaction was observed. Among these reactions, pain (40.2%), heat at the vaccination site (39.1%), erythema (36.0%) and hot abscess (25.7%) were recorded. With regard to systemic manifestations observed, the most frequently reported were diarrhea (19.0%), vomiting (19.0%), nausea (15.9%) and generalized rash (14.3%) (data are not shown in the tables).

## Discussion

The incidence rate of immunization errors had a heterogeneous distribution among the health macro-regions of the state of Minas Gerais, although the data point to an underreporting of errors. The most frequently reported type of error observed was the administration of vaccines outside minimum and maximum recommended ages, and errors without the occurrence of AEFI showed the highest incidence.

The highest proportion of reported errors was observed among children under 1 year of age. Other national studies conducted in the states of Paraná and Goiás between 2017 and 2020, and international studies carried out in Europe and the United States between 2018 and 2019, aimed to describe the characteristics of immunization errors, also found a higher incidence of errors in children under 1 year of age.^4^ ^-^ ^6^ ^,^ ^8^ ^,^ ^10^

With regard to the proportion of types of errors reported, regardless of age, almost 40% were related to the administration of vaccines outside minimum and maximum recommended ages. When comparing the results of this study with those of other studies conducted in the municipalities of Goiânia, state of Goiás, Riberão Preto, state of São Paulo, and Porto Alegre, state of Rio Grande do Sul, between 2013 and 2018, the findings regarding vaccine administered outside minimum and maximum recommended ages are similar.^6^ ^,^ ^15^ ^,^ ^16^ This type of error also occurs worldwide, as pointed out in a systematic review of the medical literature, conducted in 2019, including studies carried out in Canada, the United Kingdom, the United States, Taiwan, and Brazil.^9^

It is assumed that lack of staff knowledge and update on vaccination schedules and similarity between vaccine vials may be associated with administration of vaccines outside minimum and maximum recommended ages. An investigation conducted in the United States in 2018 also identified that vaccine schedule complexity and confusion among similar products may have contributed to administration of vaccines outside minimum and maximum recommended ages.^7^ It is important that laboratories make the necessary investment for the renewal of packaging and labelling of thier products, a key measure for greater safety at the time of vaccination for healthcare professionals.^17^

Approximately one third of the notifications of immunization errors showed unknown route of administration. National studies conducted between 2014 and 2020 in the states of Goiás,^6^ São Paulo^18^ and Minas Gerais,^19^ also found incompleteness of notification form fields, such as absence of administration route, race/skin color of vaccinated individuals and specification of the vaccine administered.^6^ ^,^ ^18^ ^-^ ^20^

The results also showed that the highest incidence of errors was related to errors without the occurrence of AEFI, corroborating those of other studies conducted in the country between 2016 and 2018.^4^ ^,^ ^6^ A systematic review of national and international studies on the prevalence of immunization errors documented between 2009 and 2018 showed that, in the majority of these studies, no adverse events following immunization errors were recorded.^9^ In the present study, the most frequently reported AEFIs were localized reactions. This fact occurs due to the act of introducing the needle causing muscle injury and irritation at the site, as well as the substances used in vaccines, such as aluminum hydroxide adjuvant, with the potential to cause an inflammatory response at the injection site.^4^ ^-^ ^6^

Despite the number of notifications observed in the study period, it is noteworthy to question the fact that less than half of the municipalities in Minas Gerais have reported immunization errors. This information suggests a hypothesis of the existence of barriers to report these incidents, possibly compatible with the difficulty of reporting due to a punitive response to errors and the lack of knowledge about the importance of reporting immunization errors, even when there is no occurrence of AEFI.^21^ ^,^ ^22^ As the filling in of information about the error is made in the same notification form for AEFI, this may contribute to an underreporting of those errors without the occurrence of adverse events, explaining - even partially - the discrepancy of the results on the incidence of immunization errors in the health macro-regions of Minas Gerais.

Other investigations, two conducted in Brazil and the United States in 2016 and one in India in 2017, also pointed to underreporting of immunization errors, which may compromise the adoption of preventive measures.^4^ ^,^ ^23^ ^,^ ^24^ This underreporting may be an indicator that there is no occurrence of errors, which contributes to its maintenance and perpetuation.^15^ Notifications should be percieved as fundamental to the safety culture, as it aims to minimize damage, in addition to fostering learning.^25^ Error reporting culture may be the first attitude towards promoting patient safety, allowing the team to feel safe and thus report the incidents,^26^ in addition to contributing to the identification of possible causes, improving the quality of care in vaccination rooms.^18^ A higher incidence of errors in some health macro-regions of Minas Gerais state is not necessarily associated with a higher occurrence, but probably to a higher notification, possibly related to an organizational culture focused on patient safety at the municipal level. Usually, errors are more exposed in institutions with a mature and strengthened safety culture.^27^

In general, immunization errors occur throughout the vaccination process, both due to failures in storage and distribution of immunobiological products, and to incorrect indication and administration to the individual.^5^ ^,^ ^9^ ^,^ ^10^ ^,^ ^28^ Lack of professionals and, consequently, work overload are factors for the occurrence of errors, which have a close relationship with work process and healthcare management.^25^

The literature has shown that the introduction of new vaccines is a contributing factor to the increase in immunization errors.^6^ ^,^ ^7^ ^,^ ^28^ This fact was evidenced during the COVID-19 pandemic, which revealed a considerable number of immunization errors, such as inadequate interval between doses, vaccine doses administered to individuals outside the recommended age group, incorrect storage and handling, among others.^29^

Supervision is an important recommendation for quality and safety in vaccination rooms. It encompasses the monitoring of the “doings” of workers and enables identifying the need for guidance and improvement, in order to prevent immunization errors.^21^ Thus, the increase in the incidence of these errors calls for greater supervision of vaccination rooms, training for health workers, risk management and direct assistance to users of the Brazilian National Health System (SUS).^6^

Another strategy to prevent immunization error lies in the involvement of the population in the process, serving as a barrier to errors. Double checking vaccines (user and professional), before their preparation and administration, should be encouraged.^5^ Using a checklist that enables verification at each stage of the vaccination process: before, during and after the administration, provides a safe preparation/ administration.^30^

Management also plays a fundamental role in preventing immunization error, providing sufficient products, inputs and human resources, given that the responsibility of developing error prevention strategies is not exclusive to health professionals. Improvement of working conditions, such as a sufficient number of workers and an adequate structure, ensure quality care for every patient and professional safety.^25^ Human nature cannot be changed, however, it is possible to improve working conditions.^21^

It is worth highlighting some limitations of this study. The use of secondary data does not allow controlling underreporting of immunization errors and the quality of information provided by SI-EAPV, which may underestimate the incidence of immunization errors in Minas Gerais. Another limitation lies in the fact that the PNI categorize as "Other" those errors that do not fit into the classification of the most common errors, which may lead to an information bias, as the frequency of this category increases. In order to minimize this bias, the most frequently reported errors, categorized as "Other", have been presented in this study.

Administration of vaccines outside the minimum and maximum recommended ages was the most frequently reported error. Immunization errors showed a heterogeneous incidence among the health macro-regions of the state of Minas Gerais, between 2015 and 2019.

The study points to a worrying scenario of immunization errors, capable of impacting on the quality of care provided in vaccination rooms, with the potential to affect the PNI, especially in a period of low vaccination coverage and growth of vaccine hesitancy. Therefore, it is necessary to encourage discussions on the need to adopt preventive measures for immunization errors.

It can be concluded that the results showed in this study can help health services in the investigation of the causes of immunization errors, supporting the adoption of preventive measures, such as the implementation of safety centers and development of patient safety plans, indispensable for safe vaccination.
